# Employing the Oncolytic Vesicular Stomatitis Virus in Cancer Virotherapy: Resistance and Clinical Considerations

**DOI:** 10.3390/v17010016

**Published:** 2024-12-25

**Authors:** Alaa A. Abdelmageed, Stephen Dewhurst, Maureen C. Ferran

**Affiliations:** 1 Biomedical Genetics and Genomics Program, University of Rochester School of Medicine and Dentistry, Rochester, NY 14642, USA; alaa_ahmed@urmc.rochester.edu (A.A.A.); stephen_dewhurst@urmc.rochester.edu (S.D.); 2 Department of Biomedical Genetics, University of Rochester School of Medicine and Dentistry, Rochester, NY 14642, USA; 3 Department of Microbiology and Immunology, University of Rochester School of Medicine and Dentistry, Rochester, NY 14642, USA; 4 Thomas H. Gosnell School for Life Sciences, Rochester Institute of Technology, Rochester, NY 14623, USA

**Keywords:** oncolysis, VSV, virotherapy, innate immunity, type-I IFN response, interferon-stimulated genes, antiviral resistance, oncoselectivity

## Abstract

Vesicular Stomatitis Virus (VSV) has emerged as a promising candidate for various clinical applications, including vaccine development, virus pseudotyping, and gene delivery. Its broad host range, ease of propagation, and lack of pre-existing immunity in humans make it ideal for therapeutic use. VSV’s potential as an oncolytic virus has garnered attention; however, resistance to VSV-mediated oncolysis has been observed in some cell lines and tumor types, limiting its effectiveness. This review provides a detailed analysis of recent advances in VSV-based oncolysis, focusing on resistance mechanisms such as sustained type-I IFN signaling, upregulation of ISGs, immune cell activation, the tumor microenvironment (TME), and tumor-intrinsic factors. Strategies to overcome resistance include enhancing viral oncoselectivity, inhibiting IFN responses, modulating the TME, and combining VSV with chemotherapies, radiation, and immune checkpoint inhibitors. Several VSV-based phase I/II clinical trials show promise; however, addressing resistance and developing novel strategies to enhance therapeutic efficacy are essential for realizing the full potential of VSV oncolytic virotherapy. Future research should focus on patient-specific approaches, as tumor heterogeneity implies varying resistance mechanisms. Personalized treatments tailored to tumor molecular profiles, along with identifying biomarkers predictive of resistance to VSV oncolysis, will enhance patient selection and enable more effective, individualized VSV-based therapies.

## 1. Introduction

Vesicular Stomatitis Virus (VSV), the prototypical member of the Rhabdoviridae family, is an arbovirus that primarily infects animals such as cattle, horses, and pigs, causing fever and fluid-filled blisters. Its genome is a negative, single-stranded RNA that contains five genes in the order 3′-N-P-M-G-L-5′, which translate into the nucleocapsid (N) protein, the phosphoprotein (P), the matrix (M) protein, and the RNA-dependent-RNA-polymerase (L). The glycoprotein (G) is embedded in the envelope and facilitates viral attachment and entry into host cells [1].

VSV has long served as a pivotal model in virology research and has been used in both in vitro and in vivo studies that led to key discoveries in viral biology, and significantly enhanced our understanding of virus–host interaction and cellular processes [2]. Due to its ease of propagation [3], relatively small genome, genetic manipulability, broad host range, and low pathogenicity and seroprevalence in humans, VSV has been used in multiple areas of research and medical applications. The VSV glycoprotein, in particular, has found utility in plasmid-based gene therapy, viral vector pseudotyping, mRNA and CRISPR-Cas delivery, and cancer immunotherapy [4].

VSV was one of the first viruses to be manipulated using reverse genetics, allowing the construction of recombinant VSV (rVSV) through targeted genome modifications [5]. This technology has paved the way for rVSV to be used as a viral vector for the delivery of therapeutic genes and the study of viral gene functions [6]. For example, the versatility of the VSV platform was demonstrated by the creation of the recombinant virus rVSV∆G-CC4, in which the G protein was substituted with the HIV receptors CD4 and CXCR4. This recombinant virus selectively targets and destroys HIV-infected cells that express the HIV attachment proteins on their cell membranes. Importantly, this virus is unable to infect uninfected cells, offering a highly specific therapeutic approach [7].

Another important and notable application of rVSV has been in vaccine development, exemplified by the FDA-approved rVSVΔG-ZEBOV-GP Ebola Vaccine (sold under the brand name ERVEBO). This is a replication-competent, live, attenuated VSV vaccine that has been genetically engineered to express the major glycoprotein gene of the Ebola virus species Zaire ebolavirus in place of the VSV G protein, and it is licensed for the prevention of disease by Zaire ebolavirus. This vaccine was successfully deployed during the 2014 Ebola outbreak in West Africa and demonstrated safety even in high-risk populations such as pregnant women [8], highlighting the potential of rVSV-based vaccines for other pathogens such as malaria and avian influenza.

Of particular relevance to this review is the development of VSV as an oncolytic agent. Cancer cells often accumulate mutations that impair their antiviral and IFN pathways, rendering them susceptible to infection by oncolytic viruses. VSV exploits these defects, selectively targeting and lysing cancer cells while sparing normal tissues. This property makes VSV a promising candidate for oncolytic virotherapy, an alternative to traditional cancer treatments that frequently lack tumor specificity and are associated with significant toxicity, relapse, and long-term adverse effects [9]. Preclinical studies have demonstrated the efficacy of VSV against a wide range of malignancies, including prostate, pancreatic, ovarian, endometrial, and hepatocellular carcinoma, as well as T-cell lymphoma, acute myeloid leukemia (AML), and melanoma. Concerns regarding VSV’s neurotoxicity can be addressed through genetic modifications, such as the substitution of the G protein or the use of attenuated mutants like MΔ51 or M51R [10], which retain oncolytic activity while improving safety profiles.

Despite its success in preclinical animal studies and early human phase I trials, resistance to VSV in certain cancer subpopulations presents a significant challenge to its therapeutic potential [11]. This review outlines key preclinical studies of VSV oncolysis and highlights recent advancements in understanding the mechanisms underlying VSV resistance in cancer virotherapy. These mechanisms include retained type-I interferon (IFN) signaling, modulation of immune cells, tumor microenvironment (TME) factors, and tumor-intrinsic characteristics. Various strategies to overcome resistance are also discussed, including enhancing oncoselectivity, blocking the IFN response, modulating the TME, addition of immune checkpoint inhibitors (ICIs), and combining VSV with adjuvant chemo- and radiotherapies. Furthermore, an overview of clinical trials evaluating the safety and efficacy of VSV-based oncolytic viruses is provided. A deeper understanding of the molecular and cellular mechanisms driving resistance, alongside the development of strategies to overcome it, is crucial for enhancing the efficacy of VSV-based virotherapy and expanding its applicability to resistant cancer subtypes.

## 2. VSV Oncolysis

Several characteristics make VSV an ideal candidate for oncolytic virotherapy. Its replication is severely limited in normal tissues that contain intact IFN responses. In contrast, VSV replicates with high specificity in cancer cells that exhibit defective antiviral signaling. This selectivity is further enhanced by the induction of IFN and cytokine genes in the TME, which boosts the immune response against tumor cells while leaving viral replication largely unaffected [12]. Another critical advantage is the low seroprevalence of VSV in humans, meaning there are no pre-existing neutralizing antibodies to impede viral replication. Moreover, VSV is not highly pathogenic in humans, typically causing a mild, self-limiting flu-like illness [13]. Potential concerns regarding neurotoxicity can be mitigated through genetic modifications, such as substituting the G with that of a non-neurotropic virus or utilizing attenuated mutants that preserve therapeutic potency while improving safety. Additionally, VSV’s rapid replication cycle enables effective lysis of cancer cells before the host antiviral response can be fully activated. Replication-competent VSV strains reach high viral titers shortly after administration, resulting in a swift oncolytic effect with relatively low doses and the release of viral progeny that can spread to neighboring tumor cells, amplifying the therapeutic outcome [14]. VSV replication is restricted to the cytoplasm, alleviating concerns about insertional mutagenesis, which is a risk with other viral vectors that can integrate into the host genome [15]. Furthermore, VSV has a broad tissue tropism, making it suitable for targeting a variety of cancer types [16]. Finally, VSV’s ease of propagation and genetic manipulability facilitate the creation of numerous recombinant strains optimized for therapeutic applications, as summarized in Table 1.

## 3. Preclinical Studies to Limit Neurotoxicity and Improve Oncolytic Efficacy

A key determinant of VSV’s oncolytic efficacy lies in its ability to exploit the defective type-I IFN signaling pathways commonly found in cancer cells, enabling selective viral replication and lysis of infected cells. In contrast, healthy cells, equipped with intact IFN responses, effectively suppress viral replication, sparing them from infection. This selective viral replication, coupled with VSV’s broad tissue tropism, has spurred significant investigation of its potential in oncolytic virotherapy [9]. Here, we summarize strategies used to limit neurotoxicity of oncolytic wild-type VSV and highlight preclinical studies that investigate innovative approaches for targeted cancer cell destruction. These studies underscore VSV’s promise as a versatile and safe oncolytic agent capable of targeting a wide range of tumor types.

WT VSV induces severe and often fatal neurotoxicity in rodents and non-human primates [28]. Therefore, possible neurotoxic effects are a critical consideration before administering VSV as a cancer treatment. Strategies to mitigate neurotoxicity include the generation of rVSV pseudotyped with an attachment protein from a non-neurotropic virus, or the use of attenuated viral strains. Additionally, VSV administration can be combined with antiviral agents that restrict viral replication within the brain [29]. Often, the strategies to reduce neurotoxicity also increase the efficacy of VSV virotherapy by increasing the selectivity of the virus for cancer cells.

### 3.1. Use of rVSV Encoding IFNβ

A promising approach to enhance oncolytic selectivity and reduce neurotoxicity involves the use of recombinant VSV encoding the IFN-β gene (VSV-IFNβ). In normal cells, VSV-IFNβ stimulates a robust innate immune response, limiting viral replication. However, in cancer cells with defective type-I IFN signaling, this immune response is impaired, allowing selective viral replication and oncolysis. For example, Jenks et al. [30] demonstrated that VSV expressing human IFN-β (VSV-hIFNβ) showed no neurotoxicity in rodent and non-human primate models of hepatocellular carcinoma. Furthermore, the inclusion of the IFN-β gene enhanced oncolytic efficiency by improving the virus’s ability to target and kill cancer cells, while also stimulating an antitumor immune response. Similarly, Patel et al. [31] reported that VSV-IFNβ effectively targeted non-small cell lung cancer (NSCLC) both in vitro and in vivo, highlighting its therapeutic potential in lung cancer treatment.

### 3.2. Use of Attenuated rVSV Encoding a Mutation at Position 51 of the M Protein

rVSV strains have been engineered to enhance therapeutic efficacy while minimizing toxicity. Notably, several strains feature a substitution (M51R) or a deletion (rVSVΔM51) at position 51 of the M protein. The modifications in these rVSVs limit their capacity to suppress the innate antiviral response, a well-characterized function of the M protein. Consequently, these engineered strains retain the ability to replicate selectively in cancer cells while reducing the risk of neurotoxic effects associated with WT VSV. Thus, these viruses present safer alternatives to WT VSV for oncolytic applications [32].

A study by Ahmed et al. [33] investigated the susceptibility of different prostate tumor cell lines to VSV. The LNCaP line demonstrated sensitivity to both WT and M protein mutant VSV, whereas the PC-3 line showed resistance. Benign prostatic epithelial cells were similarly susceptible to VSV during single-cycle infections but exhibited resistance under multiple-cycle infections due to activated antiviral responses when exposed to the M51R mutant. In in vivo experiments, M51R demonstrated less pathogenicity and complete tumor regression in LNCaP xenografts, suggesting its potential as a safer oncolytic agent for prostate cancer therapy.

Consistent with this, Ahmed et al. [19] demonstrated that mice infected with rM51R-M, a recombinant virus encoding the M51R mutation, exhibited an enhanced immune response alongside reduced neurotoxicity when compared to wild-type (WT) VSV. Both the M51R mutant and WT VSV, when administered intranasally, elicited a robust humoral IgG response due to peripheral spread. However, in contrast to WT VSV, the M51R mutant did not induce a diseased phenotype in the treated mice, highlighting its potential as a safer virus with preserved immunogenicity.

Additionally, Day et al. [34] demonstrated that VSV-M51R effectively prevented the peritoneal dissemination of colorectal cancer cells in mice following peritoneal injections of the virus. This single administration inhibited the growth of peritoneal tumors and resulted in an increase in populations of peritoneal CD4+ T cells and peritoneal cavity-resident B1b cells. Furthermore, treatment with VSV-M51R significantly reduced levels of the immunosuppressive cytokine IL-6 within the peritoneum. These findings indicate that VSV-M51R not only altered the immune and cytokine profile in the treated mice, but also conferred protection against tumor recurrence for up to 60 days post-infection, despite the limited duration of viral replication following injection.

### 3.3. Use of Attenuated rVSV Encoding a Deletion at Position 51 of the M Protein

rVSV expressing an M protein with a deletion of methionine at position 51 (rVSV-ΔM51) has also been evaluated for its oncolytic efficacy. Murphy et al. [20] investigated the ability of rVSV-ΔM51 to target and eliminate PDA cells, which represent the most prevalent and aggressive subtype of pancreatic cancer. Their findings demonstrated that rVSV-ΔM51 exhibited significant cytotoxicity against PDA cells in vitro, effectively reducing cell viability and inducing apoptosis. These cell lines were highly heterogeneous in the susceptibility to the virus. Some lines were resistant to VSV, likely due to an intact type-I IFN response. Furthermore, in vivo studies indicated that rVSV-ΔM51 not only inhibited tumor growth but also prolonged survival in murine models of PDA.

### 3.4. Use of Other Attenuated Strains of VSV

Another investigation focused on the therapeutic potential of VSVrp30, a VSV variant isolated through serial passaging on glioblastoma cells, for the treatment of systemic cancer metastasis. In this study, both human and mouse gliomas were implanted in the brains and peripheral tissues of mice. Following intravascular inoculation, VSVrp30 demonstrated selective and simultaneous lysis of multifocal brain tumors as well as subcutaneous tumors located in the flank region [22]. These findings highlight the efficacy of VSVrp30 in targeting disseminated cancer cells, suggesting its potential utility as an oncolytic agent for treating metastatic disease.

### 3.5. Use of Pseudotyped rVSV

Based on the hypothesis that the neurotoxicity of VSV is mediated by the G protein’s ability to facilitate infection of neuronal cells, several research groups have investigated the pseudotyping of VSV with glycoproteins from non-neurotropic viruses to mitigate neurovirulence. For example, Tober et al. [35] engineered a recombinant strain, VSV-GP, by replacing the VSV G protein with the glycoprotein from lymphocytic choriomeningitis virus (LCMV). This pseudotyped virus exhibited a significantly reduced neurotropism in murine models when compared to WT VSV. Furthermore, Muik et al. [36] demonstrated that VSV-GP effectively infected six out of seven prostate cancer cell lines in vitro. In mouse models of prostate cancer, both intratumoral and intravenous administration of VSV-GP led to long-term remission. Additionally, VSV-GP administration resulted in sustained remission in models of subcutaneous tumors and bone metastases, underscoring its potential as a safe and effective systemic therapy for metastatic prostate cancer. In another study focused on prostate cancer, Urbiola et al. [37] investigated the efficacy of VSV-GP in infecting various prostate cancer cell lines, as well as xenografted mouse models derived from patient samples ranging from less aggressive tumors to castration-resistant prostate cancer (CRPC). Their findings indicated substantial success, including instances of long-term remission. In a separate investigation, Schreiber et al. [38] demonstrated that VSV-GP effectively replicated and disseminated between tumors in a mouse lung cancer model, resulting in extensive destruction of cancer cells. While immune cell activation, including CD8+ T-cell trafficking, was observed, the researchers concluded that the primary therapeutic mechanism was the direct lytic activity of VSV-GP.

Finally, Wollmann et al. [39] explored a novel chimeric virus, VSV-LASV-GPC, which incorporates the glycoprotein precursor from the Lassa virus. Their results showed that WT VSV induced lethal effects following injection into the mouse brain, whereas VSV-LASV-GPC did not elicit any adverse neurological outcomes. The pseudotyped virus successfully eliminated both intratumorally injected and non-injected tumor cells, including those at the tumor periphery.

### 3.6. Combined Treatment with Antiviral Drugs

In the study by Wollmann et al. [40], the effects of antiviral agents, specifically ribavirin and chloroquine, on VSV replication in the central nervous system were investigated. Their findings demonstrated that both antiviral treatments significantly reduced VSV replication, thereby decreasing viral neurotoxicity. This study highlights the potential of these antiviral agents to preserve the therapeutic benefits of VSV as an oncolytic agent while minimizing adverse neurological effects. The results suggest that integrating antiviral drugs may enhance the safety and tolerability of VSV-based therapies, ultimately improving their application in clinical oncology.

An important goal for future studies will be to elucidate whether the antiviral drugs interfere with VSV replication to such an extent that they reduce its oncolytic activity. Another goal will be to explore the safety and efficacy of strains harboring mutations in other VSV components besides M.

## 4. Mechanisms That Drive Resistance to VSV

VSV exhibits promising potential as an oncolytic virus, but its therapeutic effectiveness can be hindered by antiviral resistance mechanisms present in certain tumors. A comprehensive understanding of these resistance mechanisms is vital to enhance the efficacy of VSV-based virotherapy. Current studies suggest that persistent activation of innate immune responses, particularly IFN signaling, plays a key role in VSV resistance. Additionally, dysregulation of specific genes and pathways, including the upregulation of ISGs (e.g., MxA, OAS) and epigenetic alterations, further contributes to this resistance [11].

To counteract these barriers, researchers are exploring innovative strategies such as combining VSV with immune modulators, chemotherapy agents, and matrix-degrading enzymes to enhance viral replication and oncolytic activity in resistant tumors. These efforts are focused on disrupting the tumor’s defense mechanisms while promoting more selective viral targeting of cancer cells. Ultimately, a better understanding of these resistance factors and the development of combination therapies will be crucial for advancing the clinical application of VSV as a cancer therapeutic.

Key mechanisms underlying resistance to VSV include the retention of type-I IFN signaling, modulation of innate cellular immunity, and modulation of the TME and tumor-intrinsic factors. These factors collectively contribute to the reduced efficacy of VSV-based oncolytic virotherapy in certain cancer types.

### 4.1. Retention of Type-I IFN Signaling

The IFN response is one of the primary obstacles to effective VSV-mediated oncolysis. In normal cells, VSV infection induces the production of type-I IFNs (IFN-α and IFN-β), which initiate antiviral signaling pathways to suppress viral replication. While many cancer cells exhibit impaired IFN signaling, some tumors retain functional IFN pathways, thereby contributing to resistance [41].

### 4.2. Constitutive Activation of IFN Response in Cancer Cells

Carey et al. [42] compared effects of VSV infection between two prostate cancer cell lines: one that is sensitive, LNCaP, and another that is resistant, PC3. They linked the resistance of PC3 cells to their retention of IFN-I responsiveness. Additionally, Bayne et al. [43] showed that the resistant prostate cancer cell line, PC3, retained an active IFN-I response, thereby constitutively expressing interferon-stimulated genes (ISGs). They attributed this constitutive activation to the expression of CHD1 and MAP3k7, two tumor-suppressor genes, which when silenced, allowed for permissive VSV-M51R infection and efficient production of viral progeny. Silencing CHD1 and MAP3k7 also decreased the constitutive expression of ISGs, emphasizing their role in the retention of an IFN-I response and the subsequent resistance to VSV.

### 4.3. Upregulation of ISGs

IFNs activate a wide array of ISGs that restrict viral replication through mechanisms such as viral RNA degradation, inhibition of protein translation, and induction of apoptosis in infected cells. Tumors capable of robustly upregulating ISGs in response to VSV are often resistant to its oncolytic effects [44]. Pavlovic et al. [13] demonstrated that VSV replication was significantly inhibited in cells expressing MxA, a key ISG, while the replication of other viruses, including two picornaviruses, a togavirus, and herpes simplex virus type 1, remained unaffected. In contrast, the expression of a related protein, MxB, did not impede VSV replication, underscoring the specific role of MxA in conferring resistance to VSV.

In a study by Moerdyk-Schauwecker et al. [45], the importance of type-I IFN signaling and ISG expression in resistance to VSV was explored. They screened 33 PDA cell lines using a VSV-ΔM51-GFP strain and found that resistant lines displayed a functional IFN-I response similar to that of non-malignant pancreatic cells. Specifically, these resistant cells constitutively expressed high levels of MxA and OAS, two key ISG proteins involved in antiviral defense. Similarly, Guo et al. [46] identified the upregulation of the Mx1 gene, MxA’s murine ortholog, as a key factor in resistance to VSV in mouse tumor models, including macrophage tumors, hepatocellular carcinoma, and melanoma. By downregulating Mx1, they observed increased VSV sensitivity, indicating that targeting Mx1 could improve oncolytic efficacy.

Using RNAseq analysis, Larrieux and Sanjuán [47] corroborated these findings in mouse melanoma cells. Their findings indicated that resistant clones exhibited chronic upregulation of ISGs, even in the absence of viral infection, suggesting a primed state of IFN-I-mediated resistance. Additionally, Huff et al. [48] identified APOBEC3 as a key gene mediating resistance to VSV in B16 melanoma cells, where its downregulation led to enhanced VSV-mediated tumor cell killing. These studies collectively highlight the role of ISGs and antiviral genes such as MxA, OAS, and APOBEC3 in mediating VSV resistance, providing potential targets for improving oncolytic virotherapy.

### 4.4. PKR-Based Mechanisms

According to Baltzis et al. [49], two eIF2α kinases—PKR and PERK—play a crucial role in mediating resistance to VSV in mouse embryonic fibroblasts (MEFs) as part of the IFN-α/β signaling cascade. Phosphorylation of eIF2α by PKR, and indirectly by PERK, leads to a global inhibition of protein synthesis, including viral RNA translation, thereby impeding viral replication.

### 4.5. Innate Immune Cells Can Impact Resistance

The immune system’s rapid response can significantly limit the spread of VSV within tumors. Natural killer (NK) cells and macrophages are key players in detecting and clearing VSV-infected cells before the virus can effectively induce tumor cell lysis. While some studies, such as Kottke et al. [50], have shown that NK cells may facilitate VSV localization within solid tumors, others report that NK cells can actually inhibit the efficacy of VSV-based cancer therapies. For instance, Altomonte et al. [51] demonstrated that NK cell-mediated clearance of infected cells can reduce the overall oncolytic activity of VSV. To address this challenge, the researchers engineered a recombinant VSV strain expressing equine herpes virus-1 glycoprotein G, which functions as a broad-spectrum chemokine binding protein (rVSV-gG); this virus has shown potential in overcoming the antiviral effects of NK cells, thereby enhancing VSV’s ability to persist and target cancer cells more effectively. These findings highlight the dual role of NK cells in VSV virotherapy and underscore the importance of modulating immune responses to optimize therapeutic outcomes.

In the study by Liu et al. [52], tumor-associated macrophages (TAMs), specifically CD68+ macrophages, were found to infiltrate ovarian and breast cancers, conferring resistance to killing by VSV. This resistance was linked to increased ISG expression, which correlated with heavy macrophage infiltration and subsequent activation of the JAK/STAT signaling pathway. This pathway, activated by low-level secretions of macrophage-derived IFNβ, established an antiviral state within the TME, preventing effective oncolysis by VSV. Anti-IFN antibodies and shRNA knock-down experiments confirmed that this macrophage-derived IFNβ was key to the resistant phenotype observed in both primary and distant VSV-infected tumors. This study underscores the critical role of TAMs in promoting resistance to VSV virotherapy through innate immune mechanisms, presenting a potential barrier to the effective use of VSV as an oncolytic agent in these cancers.

### 4.6. Tumor Microenvironment (TME) Factors That Promote Resistance

The TME plays a critical role in mediating resistance to VSV virotherapy by establishing physical and immunological barriers that hinder viral entry, replication, and immune-mediated clearance. For example, the dense extracellular matrix (ECM) of cancer cells serves as a structural barrier that impedes the dissemination of VSV within tumors, restricting the virus’s ability to effectively reach and infect target cells [53]. Moreover, Surendran et al. [54] demonstrated that adipocytes in the TME can contribute to the tumor’s resistance to VSV and that blocking fatty acid uptake by cancer cells resensitized the tumor to VSV. Several other key factors within the TME also contribute to this resistance.

#### 4.6.1. Hypoxia

Viruses generally require oxygen-rich environments for optimal replication; therefore, the low oxygen levels often found in the TME can impair VSV replication. Hypoxia also reduces the susceptibility of tumor cells to VSV infection, thereby further diminishing its oncolytic activity [55]. The hypoxia-inducible factor-1 alpha (HIF-1α), a key regulator of cellular responses to low oxygen levels, is stabilized under hypoxic conditions due to the inhibition of ubiquitination and proteasomal degradation. This stabilization of HIF-1α has been implicated in mediating resistance to VSV virotherapy [56].

Despite the initial resistance conferred by HIF-1α, Connor et al. [57] suggested that VSV might possess adaptive mechanisms to counteract hypoxia-induced resistance by enhancing viral RNA production under low-oxygen conditions. However, further studies have confirmed the role of HIF-1α in promoting resistance. For instance, Hwang et al. [58] demonstrated that HIF-1α overexpression in renal clear-cell carcinoma and glioblastoma models conferred increased resistance to VSV infection. This resistance was partially attributed to the upregulation of IFNβ, which reinforced the antiviral state within tumor cells.

Conversely, silencing HIF-1α expression using RNA interference significantly improved tumor susceptibility to VSV-mediated oncolysis. These findings underscore the dual role of hypoxia and HIF-1α in shaping the tumor’s resistance to oncolytic virotherapy and highlight potential strategies, such as HIF-1α inhibition, to overcome these barriers and enhance the therapeutic efficacy of VSV.

#### 4.6.2. Immunosuppressive Factors

The TME is often characterized by elevated levels of immunosuppressive cytokines, such as TGF-β and IL-10, as well as an abundance of immunosuppressive cells, including TAMs, myeloid-derived suppressor cells (MDSCs), and regulatory T cells [59]. These factors suppress the activation and function of effector immune cells, limiting the immune system’s capacity to recognize and eliminate VSV-infected tumor cells. These factors also play a critical role in inhibiting viral spread and oncolytic activity by obstructing T-cell maturation and limiting immune cell trafficking to the tumor site following VSV infection. Research by Weber et al. [60] demonstrated that tumor-secreted IL-6 induces CCR5 expression in a STAT3-dependent manner, resulting in increased immunosuppressive activity of MDSCs and subsequent inhibition of CD8+ T cell function in a mouse melanoma model. Conversely, a study by Diaz et al. [61] showed that the depletion of T-regs reduced the efficacy of oncolytic virotherapy, likely because it prevented the suppression of the antiviral immune response, leading to accelerated viral clearance. These findings underscore the importance of timing in the administration of therapies targeting tumor-induced immunosuppressive factors to enhance the effectiveness of virotherapy, highlighting a critical consideration for improving treatment outcomes in oncolytic cancer therapies.

### 4.7. Tumor-Intrinsic Factors That Promote Resistance

Tumor-intrinsic factors, such as apoptotic resistance and the activation of autophagy, might enable cancer cells to evade destruction by VSV while simultaneously impeding viral replication through the degradation of virions in intracellular autophagosomes [62].

#### 4.7.1. Apoptotic Resistance

Certain tumor cells exhibit mutations in pro-apoptotic genes or upregulate anti-apoptotic pathways, rendering them less susceptible to cell death induced by viral infection. A notable example is CSDE1, an oncoprotein that inhibits apoptosis and promotes metastasis. Research by Kottke et al. [63] highlighted the role of CSDE1 in cancer relapse among patients treated with rVSV-IFNβ, as it generates a neo-epitope recognized by non-tolerized T cells. To counteract this effect, the authors utilized dendritic cells engineered to express antigens against CSDE1 as a vaccination strategy. This approach significantly enhanced viral replication and increased tumor cell death in both murine melanoma and human hepatocellular carcinoma cell lines.

Similarly, Muscolini et al. [64] identified SIRT1, a member of the sirtuin family that functions upstream of p53 to inhibit its apoptotic activity as a viral restriction factor that promotes resistance to VSV oncolysis. The researchers demonstrated that knockdown of SIRT1 using miR-34a sensitized resistant PC3 prostate cancer cells to lysis by the recombinant strain VSVΔM51. Similarly, upregulation of miR-34a, achieved through treatment with histone deacetylase inhibitors (HDIs), led to decreased SIRT1 expression, which in turn enhanced both viral replication and cellular apoptosis. These findings underscore the potential for targeting SIRT1 and modulating miR-34a expression as therapeutic strategies to improve the efficacy of VSV-based virotherapy in resistant cancer cell types.

#### 4.7.2. Autophagy

Cancer cells can use autophagy as a survival mechanism against viral infection, degrading viral components before the virus can replicate effectively [65]. Nabizadeh et al. [66] identified autophagy as a tumor cell defense mechanism that can confer resistance to VSV-mediated oncolysis. They found that Beclin1, an essential autophagy gene, was expressed at low levels in VSV-sensitive HeLa cervical cancer cells, but its expression was significantly higher in VSV-resistant A549 lung cancer cells, correlating with increased autophagic activity and resistance. Importantly, silencing Beclin1 via RNA interference enhanced the sensitivity of resistant cells to VSV, suggesting that targeting autophagy pathways could improve the therapeutic efficacy of VSV-based virotherapy.

Conversely, VSV can manipulate autophagy to enhance its replication, resulting in enhanced oncolysis by autophagic death. Askari et al. [67] investigated the ability of the VSV M protein to induce autophagy-mediated cancer cell death in a chemotherapy-resistant breast cancer cell line. They concluded that both the VSV WT or M51R mutant M protein could cause autophagy-induced cell death by increasing Beclin-1 expression. Olagnier et al. [68] revealed that treatment of VSV-resistant cancer cells with the antioxidant compound sulforaphane stabilized the Nrf2 transcription factor and promoted its nuclear translocation. The resultant upregulation of antioxidant response genes (e.g., HO-1) and Nrf2-mediated autophagy suppressed the RIG-I-MAVS pathway. This disruption of the type-I IFN response enhanced VSVΔ51replication in multiple types of VSV-resistant cancer cells. Additionally, the combination of VSV with different autophagy regulators also enhanced VSV replication and oncolysis. For example, Shulak et al. [69] found that co-treatment of VSV with vorinostat, an HDI, increased VSV replication in pancreatic cancer cells by hyperacetylation of the nuclear factor-kappa B (NF-κB). This modification led to an upregulation of autophagy-related genes (ATGs), which suppressed IFN responses and enhanced VSV replication and oncolysis. In contrast, 3-Methyladenine (3-MA) treatment blocked autophagy and increased activity of ISGs, resulting in reduced VSV replication and oncolysis.

These findings underscore the need for targeted strategies that address intrinsic resistance mechanisms in tumor cells to improve the efficacy of VSV-based virotherapy and potentially overcome challenges associated with tumor-induced immune evasion.

## 5. Strategies to Overcome VSV Resistance

To counteract resistance observed in specific tumor subpopulations or metastatic lesions, numerous studies have integrated inhibitors of cellular resistance with VSV treatment. Strategies include the engineering of novel VSV constructs that exhibit enhanced replication within resistant cell types, the inhibition of IFN responses, modulation of the TME, the combination of VSV with immune checkpoint inhibitors (ICIs), as well as the co-administration of VSV with chemotherapeutic and radiotherapeutic agents. These combinatorial approaches are designed to enhance the efficacy of VSV oncolysis and diminish the impact of resistance in cancer therapy. As described in detail in Section 3 and shown in Table 1, many novel rVSVs (e.g., VSV-ΔM51, VSV-M51R, VSV-GP, and many others) have been developed to enhance oncoselectivity, reduce neurotoxicity, and enhance efficacy.

### 5.1. Use of Immunostimulatory Molecules

A study by Westcott et al. [70] compared the protective effects of exogenous IFN-α2a and IFN-β against VSV-induced cytolysis in head and neck squamous cell carcinoma (HNSCC) cells. IFN-β offered greater protection from VSV-mediated oncolysis in HNSCC cells than IFN-α2a at equivalent bioactivity levels, whereas normal cells were equally protected by both interferon subtypes. These results suggest that combining oncolytic virotherapy with IFN-α2a may enhance VSV selectivity for cancer cells, warranting further investigation into the roles of different IFN subtypes in improving the therapy’s efficacy.

Modifying VSV to express immune-modulating proteins, such as GM-CSF, IL-12, or IFN-γ, can enhance local immune responses and improve viral oncolysis. For example, Shin et al. [24] constructed a VSV recombinant expressing IL-12 and compared its infection to that of VSV-F, a fusogenic recombinant expressing a Newcastle disease virus (NDV) fusion protein, in murine squamous cell carcinoma.

Li et al. [23] investigated the oncolytic potential of a recombinant VSV featuring a Smac/DIABLO insertion between the N and M genes. This mitochondrial protein interacts with and antagonizes inhibitors of the apoptosis pathway, thus allowing the activation of caspases and apoptosis [71]. This study found that treatment with the recombinant VSV significantly reduced tumor size in mice xenografted with breast cancer cells, indicating its effectiveness as an oncolytic agent against therapy-resistant tumors.

### 5.2. Targeting Specific Tumor Mutations

Engineered VSV can be customized to selectively infect and replicate in cancer cells with specific genetic mutations. Wollmann et al. [72] examined VSV-rp30’s ability to infect and kill 19 primary human melanoma samples. They found that VSV-rp30 effectively infected and lysed most melanoma cells, even in the presence of IFN. Sequence analysis of mutations in the melanomas revealed that increased susceptibility to VSV infection correlated with mutations in BRAF, which is part of the RAS/MAPK pathway that controls cell growth, division, differentiation, movement, and apoptosis. Mutations in BRAF are found in approximately 50% of melanoma cases and correlate with an increased risk for developing brain metastases. These findings suggest that rVSV could be engineered to target cancer cells containing specific mutations.

### 5.3. Blocking the IFN Response

Combining VSV with pharmacological inhibitors of IFN signaling can mitigate oncolytic resistance in cancer cells. By using drugs that block IFN receptors, such as IFNAR inhibitors or those targeting downstream signaling components like JAK inhibitors, the IFN-mediated resistance mechanisms can be dampened. This approach potentially enhances the efficacy of VSV-based oncolytic therapies, allowing for improved targeting and destruction of tumor cells.

#### 5.3.1. IKK Inhibitors

Du et al. [73] explored the role of the upstream regulator of NF-κB, IκB kinase (IKK-β) in the IFN response following VSV infection in glioma cells. They tested the antiviral response in multiple human glioma cell lines, two of which were resistant to VSV, with and without IKK-β inhibitors. They found that IKK-β inhibitors like TPCA-1 blocked IFN-mediated gene expression, completely suppressing genes like MX1. This showed the potential synergy of combining VSV with IKK-β inhibitors for cancer treatment to overcome antiviral resistance.

#### 5.3.2. JAK Inhibitors

Numerous studies have used various JAK inhibitors to address VSV resistance. Paglino and van den Pol [44] used a JAK1 inhibitor and observed that the drug alone or in combination with the vaccinia virus B18R protein rendered the highly resistant synovial sarcoma cell line SW982 susceptible to killing by VSV-rp30a, both in vitro and in vivo. They also noted enhanced susceptibility in moderately VSV-resistant liposarcoma and bladder carcinoma cells with JAK1 inhibitor pretreatment.

To address resistance to VSV in head and neck cancer cells, Escobar-Zarate et al. [74] also employed JAK/STAT inhibitors, specifically JAK inhibitor I and ruxolitinib. They demonstrated that just one hour of exposure to these inhibitors could reverse the resistant phenotype, allowing effective viral introduction even 24 h later. Moerdyk-Schauwecker et al. [45] demonstrated that inhibiting the JAK/STAT pathway using VSV-ΔM51-GFP significantly enhanced oncolysis in resistant PDA cell lines. The treatment reduced the antiviral proteins MxA and OAS to undetectable levels in uninfected cells and substantially decreased their levels in infected cells. This reduction in antiviral defenses improved the susceptibility of PDA cells to VSV-induced cell death.

Similarly, Patel et al. [31] observed that JAK/STAT inhibition with ruxolitinib re-sensitized resistant NSCLC cells to VSV-IFNβ-mediated oncolysis. Cataldi et al. [75] used a combination of ruxolitinib and TPCA-1 (an IKK-β inhibitor) to enhance VSV-ΔM51 replication and virus-mediated oncolysis in VSV-resistant PDAC cell lines. They found that TPCA-1 was a dual inhibitor of both IKK-β and JAK1 kinase and confirmed that downregulation of type-I IFN pathways improves VSV replication and killing of resistant cancer cells. Dold et al. [76] demonstrated effective oncolytic activity of VSV-GP in ovarian cancer cell lines and xenografts, although remission in mice was generally temporary. Unlike other cancer cell lines, these ovarian cancer cells were able to produce type-I IFN, leading to an antiviral state upon viral infection. Co-treatment with the JAK1/2 inhibitor ruxolitinib reversed this antiviral response in vitro and enhanced the efficacy of oncolytic virus therapy in vivo, without significant toxicity. These findings suggest that VSV-GP combined with IFN response inhibitors may be a potent and safe therapeutic strategy for ovarian cancer.

Recently, Geoffroy et al. [77] tested the effectiveness of VSV-ΔM51 on 33 human epithelial ovarian cancer cell lines derived from patients who had failed conventional chemotherapy for ovarian cancer. The majority of these cell lines were sensitive to VSV; however, 12 out of the 33 retained a functional IFN pathway and were resistant to VSV. The addition of JAK inhibitors, such as ruxolitinib, enhanced the susceptibility to VSV in the resistant lines.

### 5.4. Epigenetic Modifications

Several research groups have explored epigenetic modulation to improve VSV replication in resistant cancer cells by using HDIs to dampen antiviral responses. Shulak et al. [69] demonstrated that treating VSV-resistant PC3 prostate cancer cells with the HDI Vorinostat induced hyperacetylation of the RELA/p65 subunit of NF-κB. This modification enhanced NF-κB activity, leading to suppression of the IFN response. As a result, VSV replication increased, and autophagy was promoted within the cancer cells, improving the virus’s oncolytic efficacy.

Nguyên et al. [78] explored enhancing VSV replication and spread in resistant prostate cancer by pre-treating tumors with the HDIs MS-275 and Vorinostat. Their study demonstrated that these HDIs sensitized VSV-resistant PC3 cells to infection by VSV-Δ51-GFP, increasing viral oncolytic activity. This enhancement was linked to suppression of cellular IFN responses and increased apoptosis. The findings suggest that HDIs can function as chemical modulators of antiviral responses and could improve the oncolytic efficacy of VSV.

### 5.5. Modulating the Tumor Microenvironment (TME)

Efforts to modify or target the TME can enhance the therapeutic efficacy of VSV in oncolytic virotherapy. Approaches such as modulating immune cells or promoting vascular normalization within the TME may reduce immunosuppressive factors, improve viral access, and increase the overall anti-tumor immune response. These strategies can optimize VSV replication, spread, and tumor lysis, thereby improving clinical outcomes.

#### 5.5.1. Immunotherapy

The ease with which VSV can be genetically manipulated allows for development of oncolytic strains designed against a particular cancer cell or tumor type. Some groups have engineered VSV to express human papillomavirus oncogene E7 [79] or human oncofetal antigen 5T4 [80], which generate CD4+ and CD8+ T cell-specific responses that traffic to the tumor site when administered intratumorally in mice with melanoma and colorectal cancer xenografts. On the other hand, Melzer et al. [81], 2018 loaded CD8+ T central memory cells with VSV in NSG mice carrying AML—thereby delivering virus directly to the tumor site via a “Trojan horse”.

#### 5.5.2. Vascular Normalization

Strategies that normalize tumor blood vessels (e.g., through anti-angiogenic therapy) can improve virotherapy by facilitating viral delivery to the tumor core. Jha et al. [82] combined rVSV-M51R with sunitinib, an anti-angiogenic drug, and observed enhanced VSV replication and cancer cell killing in vitro and a reduced tumor size in vivo in mice xenografted with prostate, breast, and renal cell carcinomas. Similarly, Alajez et al. [83] utilized a vascular disrupting agent, ZD6126, in combination with VSV-MΔ51, which resulted in increased VSV-mediated oncolysis in vitro and improved survival in an in vivo mouse model with hypopharyngeal squamous cell carcinoma xenografts.

In another study exploring vasculature normalization, Breitbach et al. [84] used an engineered VSV to selectively infect and destroy tumor blood vessels, which are crucial for tumor growth and survival. Their findings demonstrated that VSV could disrupt tumor vasculature, thereby cutting off blood supply and enhancing the virus’s therapeutic effects by limiting the tumor’s access to nutrients. Blocking thrombin activity with thrombin inhibitors has also been shown to prevent tumor vascular collapse, thereby suggesting that the approach of targeting tumor blood vessels, alongside direct oncolytic activity, presents a promising therapeutic strategy for improving the effectiveness of oncolytic virotherapy in cancer treatment.

### 5.6. Combination with Immune Checkpoint Inhibitors (ICIs)

Immune checkpoints such as PD-1, PD-L1, and CTLA-4 are critical regulators of T-cell activity, functioning to maintain immune homeostasis and prevent autoimmunity. However, cancer cells exploit these pathways to evade immune responses. ICIs targeting these molecules have therefore emerged as a therapeutic strategy, reactivating T cells to effectively target and destroy tumor cells.

VSV enhances anti-tumor immunity by inducing immunogenic tumor cell death, which releases tumor-associated antigens and promotes immune activation. Combining VSV with ICIs, such as anti-PD-L1 antibodies, synergistically enhances the clearance of tumor cells by overcoming immune escape mechanisms, significantly boosting therapeutic efficacy. This combination approach is under investigation in various clinical trials, as outlined in the Clinical Studies section.

To investigate the impact of ICI treatment on the efficacy of oncolytic VSV therapy, Cockle et al. [85] evaluated the effects of two ICIs, anti-PD-1 and anti-CTLA-4 antibodies, in a murine glioblastoma model. Mice were treated with either anti-PD-1 or anti-CTLA-4 antibodies, alone or in combination with VSV engineered to express tumor-associated antigens. The combination of ICIs with VSV significantly enhanced therapeutic outcomes, as evidenced by improved survival rates and the activation of a robust Th1 antitumor immune response. Similarly, Shen et al. [86] investigated the therapeutic efficacy of combining VSV-IFNβ-NIS with anti-PD-L1 antibodies in a murine model of acute myeloid leukemia. The combination therapy resulted in markedly increased survival and enhanced the antitumor efficacy of VSV compared to either VSV or anti-PD-L1 antibody treatment alone. These results highlight the potential for integrating ICIs with VSV-based oncolytic virotherapy to overcome immune resistance and improve therapeutic outcomes.

As a proof-of-concept, El-Sayes et al. [87] investigated the effect of type-I IFN blockade on the efficacy of oncolytic VSV therapy. Using IFNα/β inhibitors, they suppressed VSV-MΔ51-mediated upregulation of PD-L1 in mouse melanoma and adenocarcinoma cell lines. This treatment, which indirectly reduced PD-L1 expression via inhibition of the IFN signaling pathway, significantly enhanced the therapeutic efficacy of VSV. The treatment also improved T cell effector functions and increased IFNγ production, highlighting the potential of targeting IFN-I signaling to potentiate oncolytic virotherapy.

Wu et al. [88] engineered VSV to express a chimeric soluble form of the PD-L1 protein (csFV), which would effectively function as an intrinsic ICI. In preclinical studies, this engineered VSV demonstrated superior antitumor activity, leading to significant tumor growth reduction and prolonged survival in treated mice compared to controls. The therapy also enhanced anti-tumor immunity, as evidenced by an increased frequency of CD8+ effector memory T cells and CD8+/CD4+ central memory T cells.

Collectively, these studies demonstrate the therapeutic potential of combining VSV with ICIs to enhance anti-tumor immunity. By simultaneously stimulating immunogenic cell death and mitigating immune checkpoint-mediated suppression, this strategy achieves robust tumor clearance. The findings underscore the promise of VSV as a versatile oncolytic vector in conjunction with ICIs, paving the way for advanced therapeutic designs in cancer treatment.

### 5.7. Combination with Chemotherapy or Radiotherapy

Chemotherapy or radiotherapy can sensitize tumors to VSV by disrupting the tumor structure and/or inducing immunogenic cell death. Sung et al. [25] combined two VSV recombinant strains (sVSV-F and rVSV-IL12) with the potent chemotherapeutic drug cisplatin in SCC cell lines. They found that combining VSV and cisplatin enhanced viral replication and tumor cell killing. They also observed that combining cisplatin with rVSV-F reduced the tumor burden and increased survival in vivo. In contrast, they found that the combination of cisplatin with rVSV-IL12 did not enhance tumor reduction and animal survival compared with rVSV-IL12 alone. These data suggest that optimizing the use of chemotherapeutic drugs in combination with VSV may be strain/mutation-dependent. Testing another chemotherapeutic drug, gemcitabine (a nucleoside analog), Hastie et al. [89] infected five murine PDA cell lines and mice xenografted with PDA with VSV-ΔM51-GFP and found a significant reduction in tumor burden. This anti-tumor effect was further enhanced by coupling the treatment with gemcitabine.

Alajez et al. [83] tested the VSV-MΔ51 strain against the human hypopharyngeal FaDu tumor model for HNSCC in vitro and in vivo. While their in vitro data showed efficient killing of the FaDu cells and induction of apoptosis by the virus, systemic injection of virus in vivo had minimal effects on the growth of the xenografted tumors. However, tumor growth was significantly suppressed, and survival improved, when the virus treatment was combined with ionizing radiation. This suggests that combination of VSV oncolytic virotherapy and radiotherapy may have potential for treating HNSCC, which remains one of the most common cancers in the world, while also having a poor survival rate.

### 5.8. Combination with Natural Agents

Natural agents, like curcumin, have also displayed a beneficial effect when combined with rVSV. For example, in a study conducted by Fehl and Ahmed [90], co-treating prostate cancer cells with curcumin enhanced the infection and oncolytic activity of rVSV-M51R. In vivo, they noted a decrease in tumor volume in nude mice with prostate cancer. In another study, Yebdri et al. [91] observed a boost in viral replication and an increase in apoptosis of prostate cancer cell lines infected with VSV-Δ51 in combination with the natural agent triptolide. They also noted increased replication and oncolytic efficiency in a prostate cancer mouse xenograft model, and a reduction of tumor size and improved survival in a mammary adenocarcinoma mouse xenograft model.

### 5.9. Combination with Other Oncolytic Viruses

Other oncolytic viruses, like vaccinia virus, can be utilized in combination with VSV to improve the efficacy of virotherapy. Whitaker-Dowling and Youngner [92] described the inhibitory effect of vaccinia virus on the IFN-induced dsRNA-dependent protein kinase in mouse L cells. Their findings demonstrated that co-infection with vaccinia virus significantly increased VSV protein synthesis, even following IFN treatment. Le Boeuf et al. [93] further investigated the combination of vaccinia virus and VSV, and found that vaccinia enhanced VSV replication and antitumor activity, while VSV facilitated the spread of vaccinia virus within tumor cells. Additionally, Taha et al. [94] explored the combination of VSVΔ51-HER2T, an rVSV expressing a truncated HER2 to be targeted by trastuzumab, with a vaccinia recombinant expressing an HER2-targeted T-cell engager (this TCE contains an HER2-specific trastuzumab single-chain variable fragment [scFv] fused to CD3-specific scFv). The co-administration of these recombinant viruses enhanced T-cell activation, reduced metastases, and improved survival in mouse breast cancer models.

The combination of VSV with other oncolytic viruses has also been reported in several case studies. For example, Gesundheit et al. [95] employed a combination of VSV, mumps, NDV, reovirus, vaccinia, and parvoH1 viruses along with trastuzmab and tamoxifen to treat a patient with breast cancer. This multimodal approach elicited a hyperimmune response against both the primary tumor and distal metastases and resulted in a radiologically complete remission of the patient’s disease. Similarly, Forčić et al. [96] documented a unique case of a 50-year-old virologist who self-administered research-grade preparations from her own lab of a measles vaccine strain and Indiana WT VSV to treat her recurrent, muscle-invasive breast cancer. Intratumoral injections resulted in a noticeable tumor shrinkage and a recurrence-free period of 45 months following surgical resection.

Collectively, these studies provide evidence that combining VSV with other oncolytic viruses may enhance treatment outcomes. However, regulatory challenges associated with these combinatorial approaches remain a significant hurdle, necessitating further research and evaluation.

## 6. Clinical Trials

In recent years, clinical investigations have assessed the safety and efficacy of VSV as an oncolytic agent for various cancers, including lymphoma, melanoma, and neuroendocrine carcinoma (NEC). These studies encompassed multiple Phase I trials and a combined Phase I/II trial, including both dose escalation and treatment protocols.

### 6.1. Safety and Efficacy

Early-phase clinical trials have yielded evidence of a manageable safety profile and promising preliminary efficacy, although several recently initiated trials remain in progress and have yet to report final results. For example, Cook et al. [97] reported regression of lymphatic lesions in patients with relapsed refractory T-cell lymphoma following a single dose treatment with VSV- IFNβ-NIS (NCT03017820), establishing the safety of the IFNβ sodium symporter strain and its association with tumor regression. A subsequent study of the efficacy of VSV-IFNβ-NIS in combination with the PD-L1 inhibitor Avelumab has also been conducted (NCT02923466), but no results were available at the time of writing this review (https://clinicaltrials.gov/study/NCT02923466, accessed on 29 November 2024) [98].

The combination of VSV with other therapeutic modalities is also being explored in other trials. Porosnicu et al. [99] described the design of a Phase I trial of VSV-GP in combination with ezabenlimab, a PD-1-targeting monoclonal antibody. The subsequent trial is currently still enrolling patients.

Smith et al. [100] investigated VSV-IFNβ-TYRP1 in metastatic uveal melanoma through both intratumoral (IT) and intravenous (IV) administration. They also demonstrated a dose-dependent immune response to the virus encoded tumor antigen TYRP1, although there was no evidence for a reduction in tumor burden. Nevertheless, this study suggests the potential utility of VSV virotherapy in this patient population.

Most recently, McGarrah et al. [101] initiated a Phase I/II clinical trial involving VSV-IFNβ-NIS (NCT03647163), confirming treatment safety in NEC patients. In the second part of the study, VSV-IFNβ-NIS was combined with the ICI Pembrolizumab, showing preliminary indications of T-cell responses to both the virus and the tumor, thereby suggesting tumor desensitization to treatment. The concurrent use of Pembrolizumab may help mitigate the inflammatory tumor response, potentially enhancing treatment efficacy against NEC.

### 6.2. Premature Clearance: Dosing/Re-Administration

The optimal administration regimen of VSV, whether as a single or multiple doses, remains a topic of debate within the field of oncolytic virotherapy. A single dose is expected to initiate viral infection and replication within the tumor and induce neutrophil trafficking to the tumor site. However, this influx of neutrophils can concurrently limit viral replication. Notably, VSV-IFN-β has been utilized as a single-dose therapy in numerous clinical trials (NCT03647163, NCT03120624, NCT02923466, NCT03017820, and NCT04291105). Conversely, recent studies, including those by Naumenko et al. [102], suggest that small, repeated doses of VSV may overcome this and result in enhanced and sustained infection through intravascular monocytes. This experimental approach has been shown to promote CD8 T cell recruitment, delay tumor growth, and improve survival in a mouse xenograft model of colorectal carcinoma.

Overall, clinical trials employing VSV as a therapeutic agent substantiate its potential as an effective oncolytic virotherapy. Nonetheless, as outlined, addressing the challenges associated with antiviral resistance will be critical for optimizing its application in cancer therapy.

## 7. Conclusions and Future Directions

To enhance the efficacy of VSV in oncolytic virotherapy and improve its specificity against resistant cancer cell populations, a deeper understanding of its replication dynamics within these cells is essential. Although overcoming resistance remains a significant challenge, promising strategies involve the use of agents that inhibit innate immune responses in cancer cells, such as ruxolitinib (JAK1/2 inhibitor). One key observation is that cellular sensitivity to VSV-mediated oncolysis varies depending on tumor type, suggesting that cell-type-specific responses play a critical role. Consequently, future research should explore patient-specific approaches, as tumor heterogeneity implies that resistance mechanisms may differ across individuals. Personalized treatments, tailored to the molecular profiles of each patient’s tumor, will be crucial for optimizing outcomes in clinical trials.

Another promising avenue is the identification of biomarkers that predict resistance to VSV-mediated oncolysis. Biomarkers such as intact IFN signaling, elevated expression of ISGs (e.g., MxA, OAS), resistance genes like APOBEC3 or SIRT1, hypoxia markers, or high ECM cytokine expression could guide therapeutic choices and inform combination strategies. Such advancements will enhance patient selection and contribute to the development of more effective, individualized treatment protocols in VSV-based oncolytic virotherapy.

In summary, overcoming resistance to VSV-mediated oncolysis necessitates a multifaceted strategy that encompasses viral modification, disruption of resistance pathways, and the integration of combination therapies. A thorough understanding of the molecular mechanisms driving resistance is pivotal for the development of more potent therapeutic approaches. By addressing these challenges, researchers can optimize VSV-based oncolytic virotherapy, enhancing its efficacy and broadening its applicability as a promising treatment modality in cancer therapy.

## Figures and Tables

**Table 1:** Strains of VSV utilized in oncolytic virotherapy research.

WT/Modified VSV Strain	Description	Uses in Clinical/Research Studies
VSV WT-Indiana serotype [17]	One of two serotypes of WT VSV, the other being WT-NJ	Used in almost every research study that includes a WT strain of VSV
AV1 (M51R) [18]	M mutant with Met to Arg substitution at position 51	Reduced neurotoxicity and increased tumor susceptibility in prostate cancer mouse xenografts [19]
AV2 (VSV-MV221F and VSV-MS226R) [10]	M mutant with two substitution mutations: Val to Phe at position 221 and Ser to Arg at position 226	
AV3 rVSV-ΔM51 [10]	M mutant with complete deletion of the Methionine at position 51, eliminating cytotoxic effects of M	Used to infect pancreatic ductal carcinoma (PDA) cells [20]
VSV-rp30a [21]	VSV variant produced by serial passage on glioblastoma cells; replicates efficiently in glial carcinomas and sarcomas	Used to infect mice with multifocal glioma and metastatic brain carcinoma [22]
rVSV-S [23]	VSV containing a Smac/DIABLO insertion between the N and M genes (resulting in apoptosis of infected cells)	
rVSV-IL12 [24]	VSV encoding the IL12 gene to enhance tumor-cell killing	Used to treat squamous cell carcinoma (SCC) in combination with chemotherapy [25]
VSV-IFNβ [26]	VSV encoding the IFNβ gene to enhance tumor-cell killing	Used in a phase I trial in patients with metastatic uveal melanoma
VSV-IFNβ-NIS	VSV-IFNβ in addition to Sodium Iodide Symporter (NIS) to facilitate tracking of virus spread	Used to track virus spread in a phase I trial in patients with endometrial malignancy
VSV-GP [27]	VSV encoding an LCMV glycoprotein in place of VSV-G, to reduce viral neurotoxicity	Used in a phase I trial on advanced and refractory solid tumors
